# Case series of functional non epileptic seizures (PNES) in patients with brain tumors

**DOI:** 10.1007/s10072-025-08255-y

**Published:** 2025-05-31

**Authors:** Roni Loebenstein, Shany Guly Gofrit, Mailam Eltity, Erez Magiel, Uriel Fennig, Keren Altman, Ory Haisraely, Noga Atar, Elisheva Jan, Naum Margolin, Marina Boxer, Nicola Maggio, Alisa Taliansky

**Affiliations:** 1 Department of Neurology, The Chaim Sheba Medical Center, Derech Sheba 2, Ramat Gan, Israel; 2 Neuro-oncology unite, The Chaim Sheba Medical Center, Ramat Gan, Israel; 3 The Faculty of Medical and Health Sciences, Department of Neurology and Neurosurgery, Tel Aviv University, Tel Aviv, Israel; 4 Talpiot Medical Leadership Program, The Chaim Sheba Medical Center, Ramat Gan, Israel

**Keywords:** Seizures, Epilepsy, PNES, Brain tumors, Glioblastoma, Epilepsy monitoring unit

## Abstract

**Purpose:**

Functional (Psychogenic) non epileptic seizures (PNES) are events which mimic epileptic seizures without ictal electrical findings on the EEG recording during the events. Diagnosis of PNES is made by correlating the clinical presentation with the lack of ictal activity as diagnosed by video EEG (VEEG) and is crucial for proper decision-making regarding management. Patients with brain tumors have a high prevalence of epileptic seizures. However, clinician’s awareness of PNES in those patients is often insufficient.

**Patients and methods:**

We collected data regarding 6 patients with brain tumors which were followed-up in the neuro-oncology unit in Sheba medical center and were diagnosed with epilepsy, along with PNES according to VEEG.

**Results:**

We describe data regarding 6 patients aged 20–68, four of them females, with different brain tumors and in various stages of the disease. PNES were mostly focal, and a clear trigger was identified in four patients. Improvement in PNES frequency alongside a reduction in anti-seizure medicaions followed diagnosis of PNES in 5 out of the 6 patients.

**Conclusions:**

In our case series, PNES were usually focal, and patients did not have all the risk factors associated with PNES in the general population. PNES frequency was unrelated to oncological status. Diagnosis of PNES with open discussion reduced PNES frequency and dosages of anti-seizure medications in our patients.

## Introduction

Epileptic seizures are characterized by an abnormal synchronized activity of neurons. In comparison, functional (psychogenic) non epileptic seizures (PNES) are events which clinically resemble epileptic events, without an electrophysiological correlate [1], and are diagnosed by documenting the events in video electroencephalography (VEEG) in the absence of any epileptiform activity prior, during, or following the event [2]. The incidence of PNES is approximately 3.1/100,000 per year, with an even higher prevalence among patients with an established diagnosis of epilepsy. If left undiagnosed, it can lead to a high burden of unnecessary treatment, side effects and hospitalization [3]. Correct diagnosis of PNES is associated with a substantial reduction in healthcare facility use [4].

Brain tumors may cause seizures with frequencies ranging from 10 to 100% depending on tumor type and location [5]. Due to the high prevalence of seizures in patients with brain tumors, there is a relatively low threshold for epilepsy diagnosis and treatment with anti-seizure medications (ASM). PNES are probably underdiagnosed in neuro-oncology patients due to the evidence that these patients carry organic epileptogenic brain lesions.

Here, we describe six patients with brain tumors, who presented with events which were eventually diagnosed as PNES following a VEEG test in our facility. We emphasize potential risk factors and pitfalls for PNES and aim to raise awareness of this unmet need.

## Methods

File records of patients with brain tumors which were followed-up in the Neuro-Oncology unit at Sheba Medical Center during the years 2016–2024 were retrospectively evaluated. Data regarding patients with diagnosis of suspected PNES which were referred for evaluation in the in-patient VEEG unit was collected.

Patients were defined as having epilepsy according to the criteria accepted by the International League Against Epilepsy (ILAE), namely, an unprovoked seizure and a risk of at least 60% for another seizure in the next 10 years [6]. The risk for recurrent seizures included an abnormal interictal EEG [7], and presentation with seizures immediately following procedure.

Data was collected regarding demographic features; tumor type and location; seizure semiology; and psychological factors (diagnosis of depression or anxiety prior or following diagnosis, psychiatric medication prior to diagnosis). Information regarding medical status prior to the diagnosis of malignancy was collected.

### Ethical considerations

The study was approved by the Chaim Sheba Medical Center Ethics Committee (118-24-SMC). Informed consent was exempt due to the retrospective nature of the study.

## Results

### Epidemiological description

Table 1 summarizes information regarding six patients from the neuro-oncology unit which were diagnosed with PNES according to VEEG.

**Table 1:** Epidemiology, malignancy, semiology and outcome of PNES in patients with brain tumors

	Patient 1	Patient 2	Patient 3	Patient 4	Patient 5	Patient 6
Gender and Age at diagnosis of PNES	M (44)	F (27)	F (68)	F (49)	F (51)	M (68)
*Brain tumors*						
Year of diagnosis	2010	2008	2018	2015	2024	2024
Tumor type and grade	Glioma	Low grade glioma	Meningioma	Low grade glioma	Mtx non small lung cancer EGFR MUT	Glioblastoma,
	IDH1 MUT 1, co-del 1p 19q	IDH mut 1p/19q non co-del	WHO grade 2	IDH1 MUT, 1p19q NON DEL		WHO 4, MGMT MET
				WHO 3		
Tumor location	RT frontal	RT posterior frontal	LT frontal parasagital	LT insular	Bilateral including LT motor strip	LT frontal
*Findings supporting the diagnosis of epilepsy*						
First unprovoked seizure description	Focal seizure immediately following first surgery, supporting an epileptic seizure	Abnormal sensation in LT hand and leg and loss of consciousness	RT hand weakness and aphasia	The clinical presentation of the malignancy was with a focal seizure (hand paresthesia) which evolved into a generalized tonic clonic seizure.	The clinical presentation of the malignancy were events of RT hand paresthesia and weakness, with a malignancy in the LT motor strip	Appearance of suspected seizures immediately following surgery.
Interictal EEG	No epileptiform interictal activity	Presence of spike-slow wave interictal activity in RT fronto-temporal region corresponding to the lesion demonstrated on MRI.	Presence of LT temporal inter-ictal sharp wave epileptic activity.	No epileptiform activity	No epileptiform interictal activity, generalized slowing	No epileptoform interictal activity, severe LT frontal slowing
Malignancy duration prior to PNES diagnosis	11 years	13 years	3 years	7 years	1 month	3 months
Malignancy treatment lines prior to PNES diagnosis	Two surgeries, temozolamide and proton irradiation	Surgery and radiotherapy	Surgery and radiotherapy	temozolomide	stereotactic radiotherapy(SRS)	Surgery, Radiotherapy
Oncological status during PNES diagnosis	Stable	Stable	Regression	Progression	Progression	Suspected
*Seizures*						
Year of first seizure	2010	2008	Missing information	2015	2024	2024
Semiology	Abnormal movements of the left face, and hand with involuntary contractions and unclear speech lasting a few minutes without loss of consciousness.	Contractions in the left leg and hand, with the involuntary production of sound, without loss of consciousness.	1. Head turn to the right, flexion in the right hand and extension of the right leg, then a loss of consciousness and a tremor which lasts for a minute, followed by a few hours of post ictal state.	Weakness in the right hand and leg, involuntary vocalization, difficulty speaking and heavy breathing, followed by confusion which lasts for a few minutes.	Complex movement of the right wrist including flexion, extension, and repeated choreiform movements with changing amplitude, and hair brushing. At the time of the event, vocalization alone. Performing commands, with preserved consciousness.	A prior feeling of falling, with loss of consciousness and amnesia to the event. No loss of urine, tongue bite or involuntary movements. The event lasts for a minute, with full regain of consciousness after 5 min.
			2. Tremor of the lips and chin without loss of consciousness.			
ASM prior do diagnosis of PNES	clonazepam 1 mg	lacosamide 550 mg	levetiracetam 3000 mg	lacosamide 300 mg	Levetiracetam 2000 mg	levetiracetam 3000 mg
	valproic acid 1750 mg	brivaracetam 200 mg	clobazam 10 mg	levetiracetam 3000 mg	lacosamide 600 mg	lacosamide 100 mg
	brivaracetam 400 mg	clobazam 60 mg	carbamazepine 400 mg		clobazam 10 mg	
ASM following diagnosis of PNES	clonazepam 0.75 mg	lacosamide 400 mg	levetiracetam 1250 mg	brivaracetam 200 mg	lacosamide 200 mg	levetiracetam 2000 mg
	valproic acid 1740 mg	brivaracetam 200 mg	clonazepam 1 mg	lacosamide 300 mg	clobazam 10 mg	
	brivaracetam 200 mg	clobazam 60 mg				
	lacosamide 200 mg					
PNES frequency prior to PNS diagnosis	Once a month.	Up to 50 times a day	No known frequency	1–4 times per day	1–2 times per day	1 time per week to 1 per 2 weeks, in the two weeks prior to the VEEG addmission, an event every day
PNES frequency following PNS diagnosis	Since 2022 (a year following the diagnosis) seizure free.	Cessation of events.	No events in the first two years following PNES diagnosis, with one event after being told about tumor progression.	General improvement in well-being, with reduction in events (no documentation of exact number).	Reduction of frequency to two events in 3 months.	Frequency improved until Cessation.
Emergency referal due to seizure prior to diagnosis 1 year	1	1	9	2	1	3
Emergency referal due to seizure following diagnosis 1 year	0	0	1	lack of documentation	0	4
*Psychological and psychiatric factors*						
Psychological treatment following diagnosis	Yes, following diagnosis of malignancy	Yes	multidisciplinary rehabilitation treatment	Yes, following PNES diagnosis	Yes, following PNES diagnosis	Follow-up in the supportive care unite
Additional information	Is currently in the process of lowering dose of ASM	Reduction of ASM following diagnosis of PNES, and treatment initiation with SSRI	Ruction of ASM following diagnosis of PNES	No changes in ASM	Reduction of ASM following diagnosis of PNES, and treatment initiation with SSRI	Ruction of ASM following diagnosis of PNES
Psychiatric diagnosis following malignancy diagnosis	Anxiety disorder nos	No	Panic disorder	No information	No information	No information
			anxiety disorder			

### Patient 1

A 44 y/o male diagnosed with right frontal glioma at the age of 33. Had focal seizures following brain surgery with semiology of left face and hand contraction and unclear speech with preserved awareness. 11 years following the diagnosis of malignancy, PNES were diagnosed. This was in temporal proximity to a stressful situation which was related to his spouse. Following PNES diagnosis, received a psychological treatment and had his ASM lowered dosage without exacerbation of seizures.

### Patient 2

A 27 y/o female diagnosed with a right posterior frontal low grade glioma at the age of 14. She had seizures with abnormal sensation in the left hand and leg with loss of awareness and an EEG documentation of interictal spike and slow wave activity corresponding to the lesion. In the years to follow she had events of contractions of the left side of her body with vocalization and preserved awareness. PNES were diagnosed 13 years following malignancy diagnosis, around the time of the deaths of a number of friends due to their malignancy with higher frequency in close temporal proximity to her engagement. Following PNES diagnosis, seizure frequency and ASM dosage were reduced.

### Patient 3

A 68 y/o female diagnosed with left frontal parasagittal meningioma at the age of 65. She had seizures of involuntary movement of the right hand and aphasia with impaired awareness. She also had evens of shaking and lips movement with preserved awareness which worsened upon mantle stress. She had an EEG finding of interictal discharges of left temporal sharp waves. PNES were diagnosed 3 years following malignancy diagnosis. She was treated by a multidisciplinary team with reduction in seizure frequency and ASM. However, she did not remain seizure free.

### Patient 4

A 49 y/o female diagnosed with left insular low-grade glioma at the age of 42. Her malignancy presented with a focal seizure manifesting as hand paresthesia with evolution to bilateral tonic clonic seizure. She also had events of right-side paresis and confusion accompanied by heavy breathing. PNES were diagnosed 7 years following malignancy diagnosis. The diagnosis was in temporal proximity to a death of a sister. She was treated by a psychologist with a reduction in events frequency, and with changing of her ASM combination.

### Patient 5

A 51 y/o female diagnosed with bilateral metastasis of lung cancer at the age of 51. Her malignancy presented with right hand paresthesia and weakness. She later developed repeated choreiform movements of the right hand and wrist, and vocalization, with preserved awareness. PNES were diagnosed one month later. She was treated by a psychologist with a reduction of events and ASM.

### Patient 6

A 68 y/p male diagnosed with a left frontal glioblastoma with seizures immediately following brain surgery. He later developed events with prior sensation of falling, followed by a loss of consciousness with no anormal movements, returning to his baseline state after 5 min. PNES were diagnosed 3 months following malignancy diagnosis. The patient was followed in the supporting care unit. The frequency of PNES gradually reduced until they stopped.

### Diagnosis of epilepsy

All patients were diagnosed with epilepsy, based on the presence of at least one unprovoked seizure together with an interictal EEG activity (patients 2 and 3), a first seizure immediately following a procedure (patient 1, 6), or as a presenting symptom (patients 4,5) along with a significant structural lesion determined as epileptogenic and fitting seizure semiology (Table 1).

### Malignancy and PNES

In all cases (excluding one case in which a very recent diagnosis prevented sufficient follow-up time) seizures were reduced following PNES diagnosis, and in 5/6 patients it resulted in ASM reduction. All patients received some form of rehabilitation treatment including psychological treatment and supportive care unit follow-up. Although during the VEEG monitoring all patients had suspected events without an electrographic correlation, two of them had an epileptiform inter-ictal activity. The others had seizures suspected to be of an epileptic origin due to their timing, immediately following surgery or as a presenting symptom of their malignancy (Table 1).

## Discussion

Here we describe our experience with six patients with brain tumors which were diagnosed with PNES (in addition to their epileptic seizures) following VEEG. The phenomenon of PNES in oncological patients was described in the literature by Morrow as a possible pitfall in the use of seizure rate as a glioma response parameter [8]. It was later described by Garcia et al., with a cohort of 4 patients, 3 of which had their malignancy diagnosed following a seizure, and none of them had a progression of their malignancy during diagnosis of PNES (resembling some of our cohort) [9]. Sumangala et al. presented 9 patients with a semiology similar to our cohort [10]. Our results present VEEG evidence of PNES in all the patients in this cohort, thus substantiating the PNES diagnosis, enabling a clear characterization of these patients.

There are a few differences between our population risk factors and those of the general population diagnosed with PNES. PNES are most common in ages 25–44, with a low occurrence rate in individuals older than 55 [11]. Our cohort included two older patients, in which the stress factor (diagnosis of malignancy) occurred in their late 60’s. Females are more prone to PNES [12], which was evident in our cohort as well.

Risk factors for PNES reported in the literature include anxiety, depression, childhood neglect, stress, and neuropsychological underperformance [13]. Patients with brain tumors are reported to have higher rates of depression, anxiety and cognitive decline [14]. Combined, these factors put patients with brain tumors at very high risk for developing PNES. Interestingly, patients in our cohort were diagnosed with PNES in all stages of their oncological disease, including stable disease, progression and regression, a finding previously reported in this population [9]. Of note, four of the patients in our cohort had a significant family related stressor during the events of the PNES, which was not directly related to their oncologic status.

PNES manifest in the general population mostly by generalized motor seizures (48%), whereas focal motor seizures account for only 5.9% of PNES [15]. In our cohort five patients had focal events (with and without impaired awareness). This common presentation of PNES in patients with brain tumor was previously described [8, 10].

Time to PNES diagnosis in the general population varies between studies, and is usually long, up to 3.8 years for patients with co-occurrence of epileptic seizures [16]. Head trauma was found to be associated with a late diagnosis as a possible confounder due to its association with post traumatic epilepsy [17]. In our cohort, time from malignancy diagnosis to PNES diagnosis shorten during the years, with rising awareness to this phenomenon and the addition of a neurologist to the provider caring for these patients.

In the general population, frequency of PNES reduces following diagnosis, with up to half of the patients becoming seizure free [18]. In our cohort, all of the patients had a reduction in PNES following diagnosis, and in (3/6) there was seizure freedom, stressing the importance of communicating the diagnosis to the patient as part of the treatment. The higher rates of PNES reduction following diagnosis in patients with brain tumors compared to the general population calls for future research regarding the difference between the two populations, including lack of known risk factors for PNES in the first.

Our study has several limitations. VEEG was not performed routinely, but rather in cases of high clinical suspicion for PNES, which include lack of improvement in seizure frequency with raising dosages of ASM, and with lack of correlation to oncological status, as well as semiology which is not explain by tumor location. Future systematic research to evaluate the extent of this phenomenon needs to consider this possible selection bias.

To conclude, in our experience, PNES does not correlate with oncological disease course, and known PNES risk factors can be missing. The presentation of PNES in our patients with brain tumors is different from the general population, being mostly focal. High index of suspicion, and early evaluation with VEEG is warrant in suspected cases, including in patients with no prior psychiatric disease. Discussing the diagnosis of PNES with the patient, and the appropriate psychological interventions are probably the reasons for the reduction of events documented in our patients. Further research is needed to collect data regarding the epidemiology and prognosis of patients with PNES and malignancy.

## Data Availability

All data generated or analysed during this study are included in this published article.
